# An Exponential Regression Model Reveals the Continuous Development of B Cell Subpopulations Used as Reference Values in Children

**DOI:** 10.3389/fped.2018.00121

**Published:** 2018-05-04

**Authors:** Christoph Königs, Stephan Schultze-Strasser, Andrea Quaiser, Konrad Bochennek, Dirk Schwabe, Thomas E. Klingebiel, Ulrike Koehl, Claudia Cappel, Udo Rolle, Peter Bader, Melanie Bremm, Sabine Huenecke, Shahrzad Bakhtiar

**Affiliations:** ^1^Department of Pediatric and Adolescent Medicine, University Hospital Frankfurt, Frankfurt, Germany; ^2^GMP Development, Integriertes Forschungs- und Behandlungszentrum Transplantation (IFB-TX), Hannover Medical School, Institute of Cellular Therapeutics, Hannover, Germany; ^3^Department of Pediatric Surgery, University Hospital Frankfurt, Frankfurt, Germany

**Keywords:** B cell subpopulations, reference values, exponential model, autoimmunity, flow cytometry

## Abstract

B lymphocytes are key players in humoral immunity, expressing diverse surface immunoglobulin receptors directed against specific antigenic epitopes. The development and profile of distinct subpopulations have gained awareness in the setting of primary immunodeficiency disorders, primary or secondary autoimmunity and as therapeutic targets of specific antibodies in various diseases. The major B cell subpopulations in peripheral blood include naïve (CD19^+^ or CD20^+^IgD^+^CD27^−^), non-switched memory (CD19^+^ or CD20^+^IgD^+^CD27^+^) and switched memory B cells (CD19^+^ or CD20^+^IgD^−^CD27^+^). Furthermore, less common B cell subpopulations have also been described as having a role in the suppressive capacity of B cells to maintain self-tolerance. Data on reference values for B cell subpopulations are limited and only available for older age groups, neglecting the continuous process of human B cell development in children and adolescents. This study was designed to establish an exponential regression model to produce continuous reference values for main B cell subpopulations to reflect the dynamic maturation of the human immune system in healthy children.

## Introduction

B lymphocytes are key players in humoral immunity, expressing diverse surface immunoglobulin receptors directed against specific antigenic epitopes. Their maturation originates in the bone marrow, where they rearrange their immunoglobulin light and heavy chain genes, and is further continued in secondary lymphoid tissue (1). In peripheral blood, B cells can be divided into CD27^−^IgD^+^ naïve, CD27^+^IgD^+^ non-switched memory and CD27^+^IgD^−^ switched memory B cells (2). In addition to this functional classification of B cells, which is based on the state of B cell maturation, further B cell subsets, such as plasmablasts (CD24^hi^CD27^int^), and B10 cells (CD24^hi^CD27^+^), immature cells (CD27^hi^CD38^hi^) and Br1 cells (CD25^hi^CD71^hi^), which all belong to the group of regulatory B cells (Breg), have received increased awareness during the past decade (3). In addition to pathogen defense, B cell subsets are known to have a suppressive function, especially through interleukin 10 production, and are involved in maintaining self-tolerance, especially in the interactive balance of B and T cell interplay in the germinal center (3).

An impairment of B cell numbers and/or function has been described as the underlying pathology in various primary immunodeficiency disorders (PID) (4, 5). The so-called common variable immunodeficiency (CVID) is the most common PID in adults. The current gold standard for CVID classification, EUROclass, separates two groups of patients according to their B cell numbers, further dividing them into subgroups based on the numbers of memory B cells and transitional B cells in the peripheral blood (6). CVID often results in a reduction of switched memory B cells (7–10). A recent development in genetic analysis has identified a series of monogenetic disorders among CVID patients, breaking this PID pool down to several monogenic disorders, mostly with severe courses of disease that also affect children at young ages (11). These include hyper IgM syndromes (12), ICOS- (13), CD19- (14), CD20- (15), CD81- (16) and BAFF-receptor- (17), LRBA-deficiency (18), CTLA-4-haploinsufficiency (19), and PI(3)K delta deficiency (20), and the list is growing. While affected patients can receive immunoglobulin G replacement by regular intravenous or subcutaneous infusions, they lack other isotypes and B cellular functions. These patients suffer primarily from the consequences of B cell dysregulation, such as multi-organ autoimmunity or malignancy (10, 21).

The flow cytometric analysis of B cells has become an important diagnostic tool in the classification and understanding the underlying pathology in patients suffering from PID (22, 23). Reliable B cell subtype phenotyping is also necessary for the development and evaluation of targeted anti-cellular therapies, such as rituximab (24), bortezomib (25), and daratumumab (26) for patients with refractory B cell and antibody driven autoimmunity, especially hemolytic anemia. Additionally, a detailed analysis of B cells is of utmost importance in the reconstitution phase after allogeneic stem cell transplantation (alloHSCT) and/or following cellular therapies such as chimeric antigen receptor (CAR) T cell therapies.

B cell composition, however, is strongly age-dependent. Therefore, age-matched reference values are a prerequisite for the successful identification of patients with B cell abnormalities, especially for patients with clinical disease onset during early childhood. Regarding B cells, few studies have provided age-matched pediatric reference values by the analysis of CD20^+^ or CD19^+^ B cells in the peripheral blood of children and adults in the 5–7 age group (23, 27, 28). Nevertheless, data on normal values in age groups only reflect the dynamics of natural B cell development in a very limited fashion. The application of age groups is associated with steps in the resulting reference values, not correctly representing the continuous process of human B cell development. Thus, the introduction of age-matched reference values for the major lymphocyte subpopulations based on exponential regression models overcomes these artificial steps. In earlier studies, such models have been implemented for major lymphocyte and dendritic subpopulations (29, 30). Regarding B cells in pediatric patients, we are aware of only one study using an exponential model of reference values in pediatric patients in five age groups (31).

Herein, we present a large pediatric cohort with an exponential model ensuring close age-correlated reference values for B cells among different ages to reflect the dynamic maturation of the human immune system in children.

## Materials and methods

### Study cohort

Between June 2010 and January 2014, 227 healthy children were enrolled in this cross-sectional, monocentric study. Clinical or laboratory parameters were determined to exclude study participants with acute infections or immunodeficiencies (e.g., clinical symptoms of PID, CRP and counts of leukocytes, lymphocytes and neutrophil granulocytes according to age). Inclusion criteria were age <18 years, no clinical signs of acute infections or incidence of immunodeficiency (defined as: >2 severe infections per year, >8 infections per year, persistent fungal infections, failure to thrive or post-vaccinal complications, no evidence of acute bleeding, negative CRP and normal cell count). Residual blood samples of otherwise healthy children who received pre-surgery work-ups were used for this study according to the guidelines of the medical ethics committee of Frankfurt University Hospital (IRB approval, Ref. No.139/09).

### Blood sample preparation and flow cytometry

Peripheral blood (PB) samples were collected using EDTA tubes and were analyzed within 24 h after collection. Cells were washed twice with PBS and stained with different combinations of monoclonal antibodies (MoAb) against CD45, CD19, CD20, anti-IgD, and CD27 and incubated in the dark for 15 min at room temperature. The MoAbs were conjugated with fluorescein isothiocyanate (FITC), phycoerythrin (PE), phycoerythrin-Texas-Red-Tandem (ECD), phycoerythrin-cyanin-5 (PC5) or phycoerythrin-cyanin-7 (PC7). All reagents were obtained from Beckman Coulter (Immunotech, Marseilles, France) except anti-IgD (BD Pharmingen™, Oxford, UK). For erythrocyte lysis, 1 ml of NH_4_Cl-lysis was added to the samples and incubated for 10 min. Measurements were directly performed on a FC500 five-color flow cytometer (Beckman Coulter, Krefeld, Germany).

**Figure 1:**
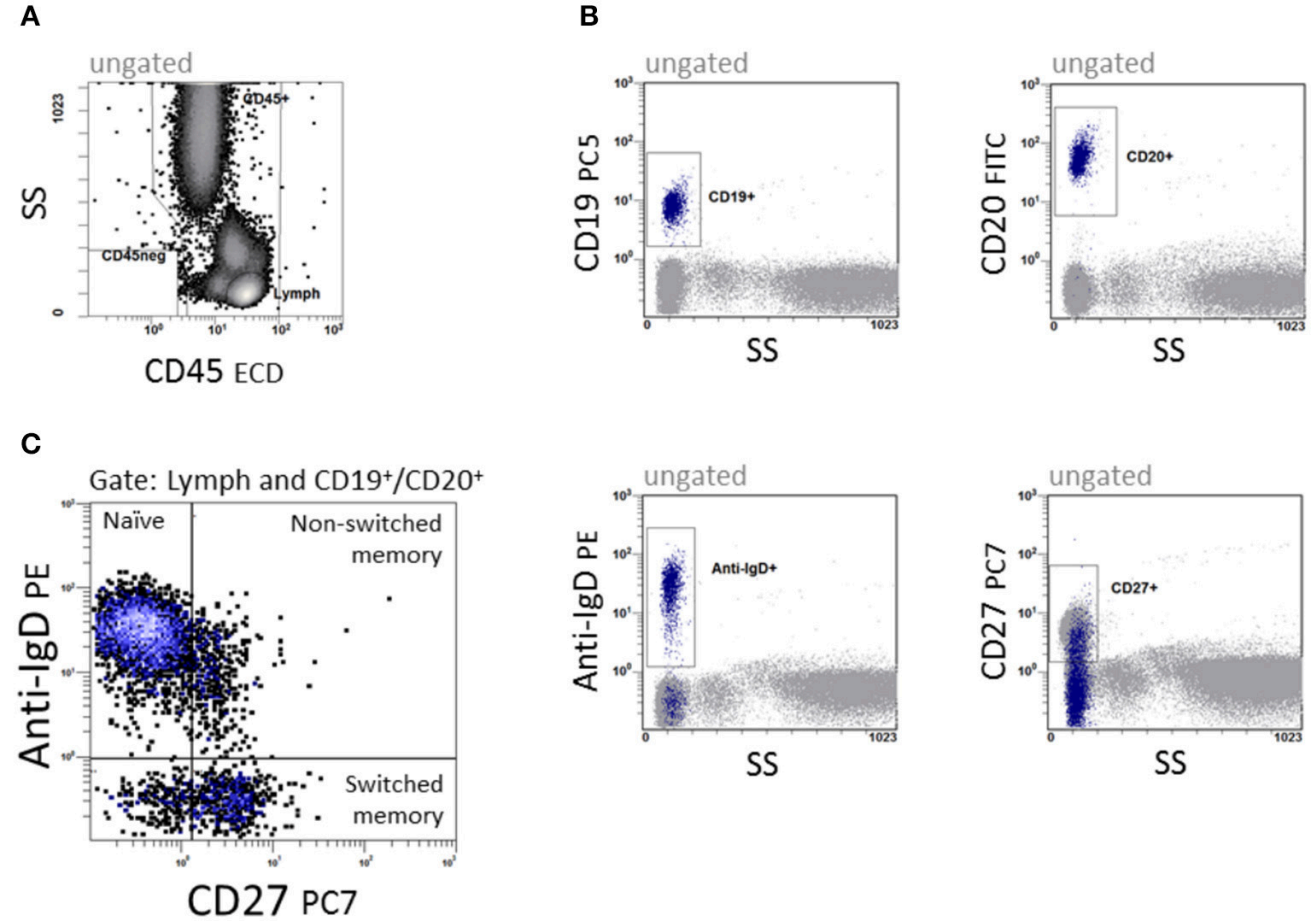
Quantification of B cell subpopulations. In a 5-color panel, surface labeling of monoclonal IgG1 and IgG2a antibodies against CD45, CD19, CD20, anti-IgD and CD27 were conjugated with fluorescein isothiocyanate (FITC), phycoerythrin (PE), phycoerythrin-Texas-Red-Tandem (ECD), phycoerythrin-cyanin-5 (PC5) and phycoerythrin-cyanin-7 (PC7) for staining. **(A)** CD45 vs. side scatter (SS) to define the lymphocyte region. **(B)** The four dot plots were gated on CD45^+^ cells and give an overview of the antigen expression of CD19, CD20, Anti-IgD, and CD27. **(C)** CD27 vs. anti-IgD to define CD19^+^/CD20^+^IgD^+^CD27^−^ naïve, CD19^+^/CD20^+^IgD^+^CD27^+^ non-switched and CD19^+^/CD20^+^IgD^−^CD27^+^ switched memory B cells.

Immunophenotyping of B cell subsets was performed in a dual platform approach. The quantity of CD19^+^ and CD20^+^ B cells was analyzed, and subpopulations were related proportionally to CD19^+^ and CD20^+^ B cells, respectively. B cells were differentiated into naïve (CD19^+^ or CD20^+^IgD^+^CD27^−^), non-switched memory (CD19^+^ or CD20^+^IgD^+^CD27^+^) and switched memory B cells (CD19^+^ or CD20^+^IgD^−^CD27^+^) as shown in Figure 1. Data were analyzed with CXP v2.2 software (Beckman Coulter).

### Quality control

The optical alignment and fluidics stability of the flow cytometer were tested with Flow Check Pro Fluorospheres (Beckman Coulter, Brea, USA) daily before measurements. Immuno-Trol™ (Beckman Coulter, Brea, USA) was used and evaluated for verification. Flow set Fluorospheres (Beckman Coulter, Brea, USA) were used to set up the photomultiplier tube values weekly. In addition, stained Cyto-Comp cells (Beckman Coulter, O'Callaghan's Mills, Ireland) were used to compensate for the fluorescence overlap.

### Statistical analysis

To examine B cell development during childhood and adolescence in a continuous regression manner, a three-parameter non-linear exponential model, initially published by R.J. Oosterbaan in 1994, was chosen to describe the dependence of the cell count results concerning age. The model was fitted to the data by minimizing the sum of squares of the residual values. The residual values of this model were used to define reference intervals with confidence probabilities of 0.90 and 0.95. The non-linear regression did not make an assumption about the distribution of the variable for which the reference interval was computed. The reference intervals were added to the predictive values provided by the exponential regression function, thus creating reference intervals that are adjusted to age. Diagrams were generated with GraphPad Prism 5 (GraphPad Software, San Diego, CA, USA).

## Results

### Study population

Between June 2010 and January 2014, 227 patients were assessed, of whom 43 did not meet the inclusion criteria, due to abnormal blood cell counts or positive CRP (*n* = 16), or missing lab parameters necessary for inclusion (*n* = 21) or due to insufficient material for flow cytometry (*n* = 6). A total of 184 children aged from 2 days to 18 years, 64 females and 110 males, met the inclusion criteria. Children and adolescents were enrolled according to their age, leading to 15 in the age group 0–12 months, 21 in 12–24 months, 57 in the age group 2–5 years, 37 in the age group 6–12 years and 42 in the age group 13–17 years.

### Reference calculator

**Figure 2:**
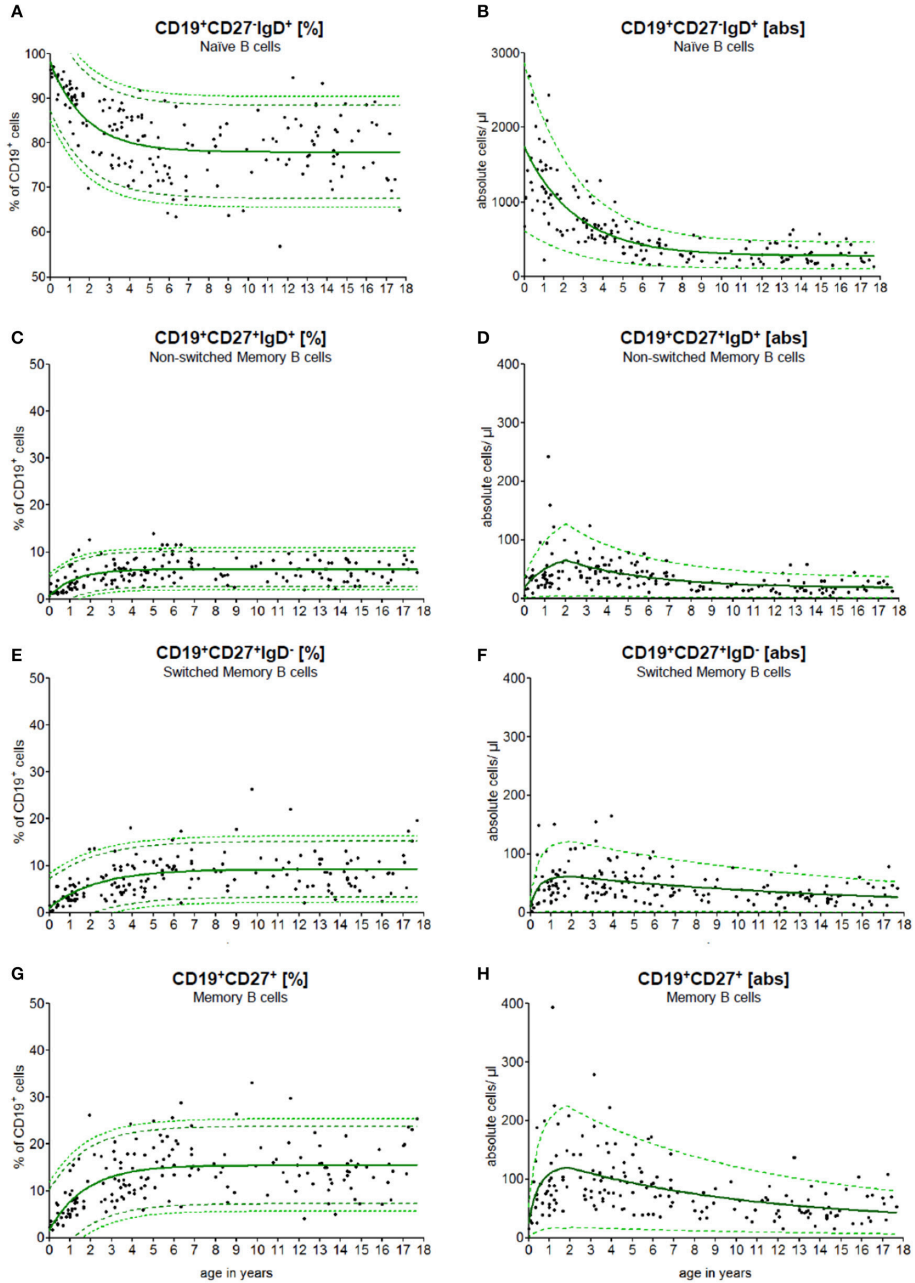
Age-matched reference values of B cell subpopulations. The continuous exponential regression model shows the relative frequencies **(A**+**C**+**E**+**G)** and the absolute cell count **(B**+**D**+**F**+**H)** of B cell subpopulations in the peripheral blood of healthy children. The black dots (•) are the raw data of every single measurement. The green solid line (—) defines the predictive value, the green dashed line (- -) represents the 90 % and the green pointed line (···) represents the 95% confidence level of the upper and lower limit.

To use the continuous reference values for diagnostic purposes, we implemented a calculator that computed the reference range for naïve, switched memory and non-switched memory B cells for patients at different ages (Figure 2). The data have been integrated into the local laboratory system to allow for the direct interpretation of physiological and pathological results.

### Reference values of B cell subpopulations

PB samples were prepared and stained as described in methods. An example of staining and data acquisition is shown in Figure 1. Data of the normal healthy children were not classified into arbitrary age groups. Raw data were calculated into a non-linear exponential regression model. The calculated regression model for the absolute cell count and the relative frequency of the B cell subpopulations is shown in Figure 2. As expected, the major B cell population (in median 1,800 cells/μl and 98% of CD19^+^ B cells) at birth expresses CD19^+^CD27^−^IgD^+^ and belongs to the naïve phenotype (Figures 2A,B). The naïve B cell subpopulation decreased strictly in the first 10 years and remained stable until the age of 18 years. The percentages of non-switched, switched and memory B cells increased notably within the first 5 years and remained stable thereafter (Figures 2C,E,G). The absolute cell counts of non-switched, switched and memory B cells displayed a short-term increase with a peak at the age of 2 years and then a slow-going decrease (Figures 2D,F,H). The minimal absolute cell counts of non-switched, switched and memory B cells in children over 6 months were 5, 8, and 16 cells/μl, respectively.

**Figure 3:**
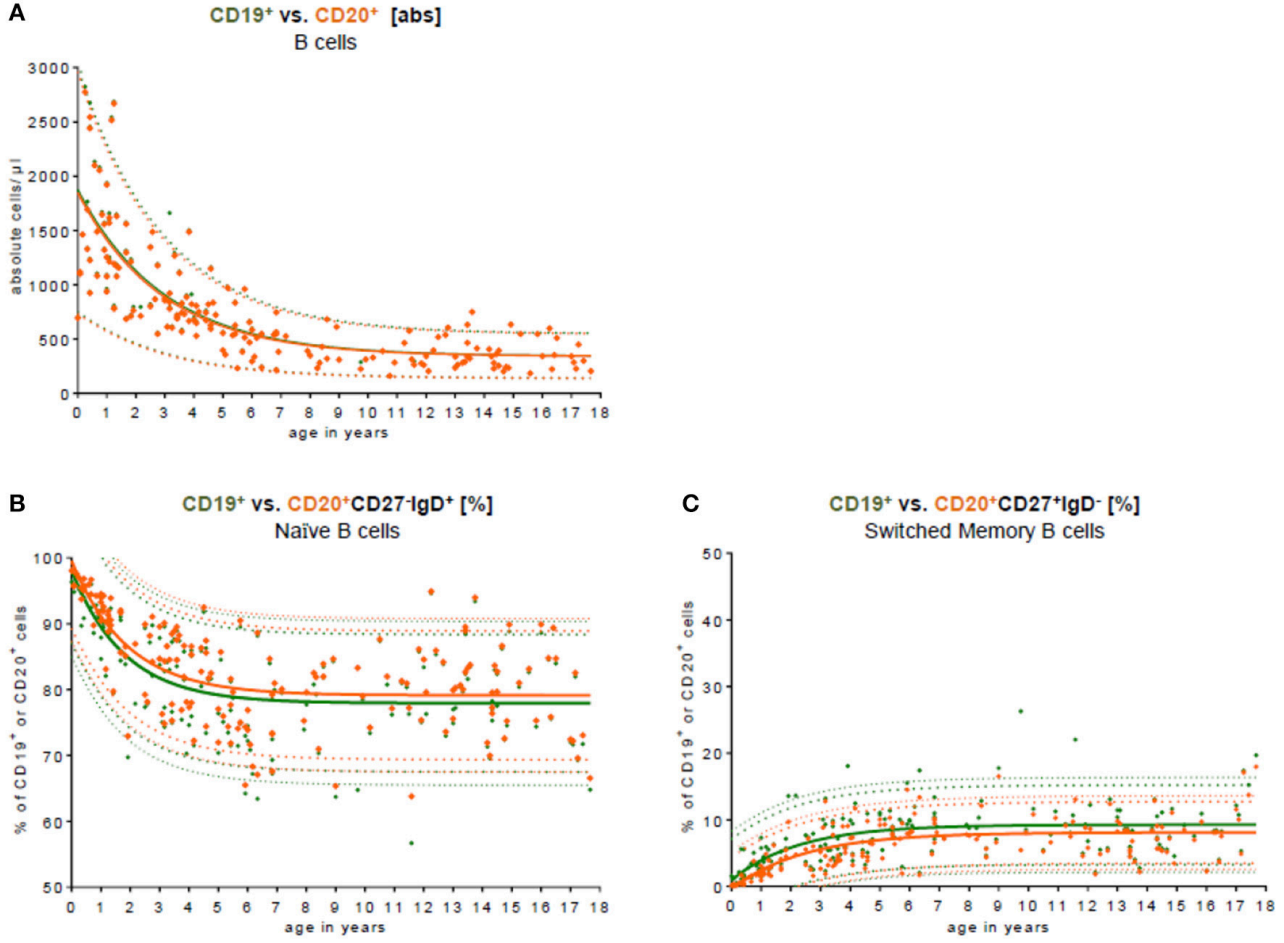
Comparison of CD19 and CD20 as key B cell markers. **(A)** The absolute cell count of CD19^+^ B cell (green) vs. CD20^+^ B cells (orange) in the peripheral blood is shown. The median difference in the absolute cell count was 7 cells/μl (0–131 cells/μl). There was no significant difference in the peripheral blood. Approximately 99.0% of all CD19^+^ B cells co-expressed CD20. **(B)** The relative frequency of naïve CD27^−^IgD^+^ B cells is shown in relation to CD19^+^ and CD20^+^ B cells, respectively. The relative frequency of naivety was around 2% lower using CD19 as a key B cell marker. **(C)** The relative frequency of switched memory CD27^+^IgD^−^ B cells is shown in relation to CD19^+^ and CD20^+^ B cells, respectively. The relative frequency of switched memory B cells was around 2% higher using CD19 as key B cell marker.

To test the conformity and to compare our data with other studies addressing differences in CD19 and CD20 expression as the key B cell marker in the peripheral blood, we used both markers (Figure 3). Approximately 99.0% of all CD19^+^ B cells co-expressed CD20. The median difference in absolute cell count was 7 cells/μl (0–131 cells/μl). There was no significant difference in B cell numbers when CD19 was used for total C cell numbers compared to CD20 (Figure 3A). When looking at the relative frequency of B cell naivety, it was approximately 2% lower in the subpopulation using CD19 as key B cell marker (Figure 3B). For switched memory B cells, the relative frequency was, in contrast, around 2% higher in using CD19 as a key B cell marker (Figure 3C).

### Age-based reference values

**Figure 4:**
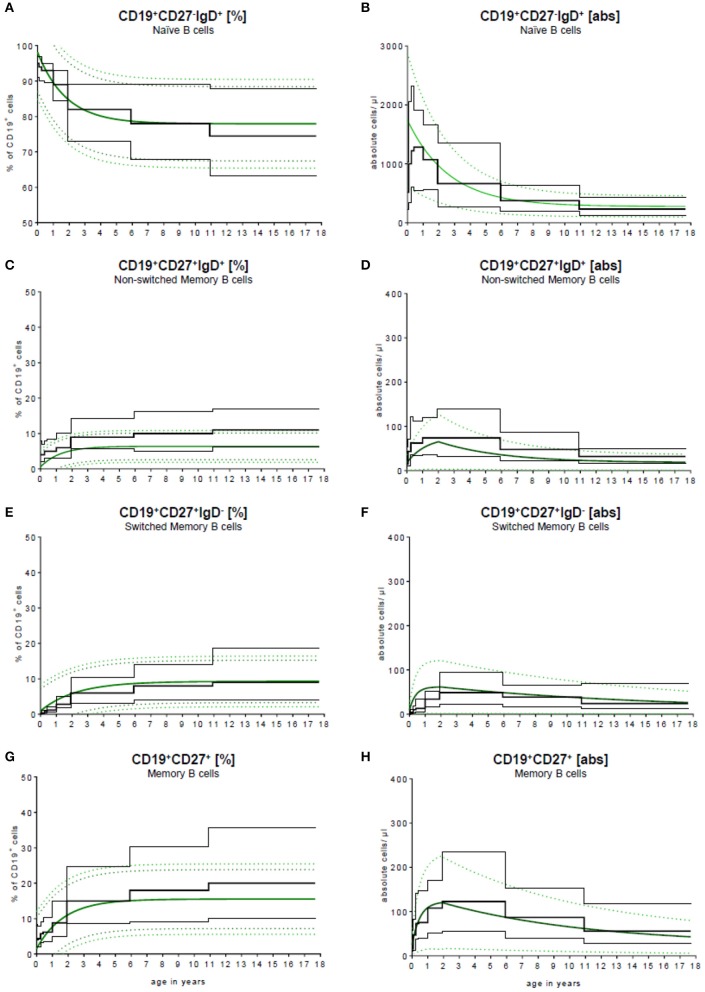
Comparison of our model with published data. Our continuous exponential regression model showing the relative frequencies **(A**+**C**+**E**+**G)** and absolute cell counts **(B**+**D**+**F**+**H)** of B cell subpopulations in the peripheral blood compared to the data from literature. Black lines indicate the 10th and 90th percentiles and the medians previously described in Huck et al. (23) with *n* = 166.

To validate our model, we compared the results published by Huck et al. (23) with our results. Huck et al. defined the group by the 5th, 10th, 90th, 95th percentile, median and mean of 166 healthy children in 5 age groups and classified B cell subpopulations through CD20. To compare these data with our data, they were fitted into our continuous regression model (Figure 4). While the age groups of Huck et al. showed sudden increases and resulted in large differences between adjacent age groups, our regression model reflected the continuous development of B cell subpopulations in childhood with a smooth bridging of age reference levels. The most pronounced difference was seen in the frequency of non-switched memory (Figure 4C) and memory B cells (Figure 4G). Here, Huck et al. presented more non-switched memory B cells (10th to 90th percentile in the age group 11–18 years) with 6.1–16.9%. In contrast, the range in the same age was 2.6–10.2% in our model. Accordingly, in the memory B cell compartment (summation of switched and non-switched B cells, age group 11–18 years), the range from the 10th to 90th percentile was 10.2–35.6%, contrary to 7.3–23.6% in our model. Despite that, the differences in the absolute cell count were only marginal (Figures 4B,D,F,H).

**Figure 5:**
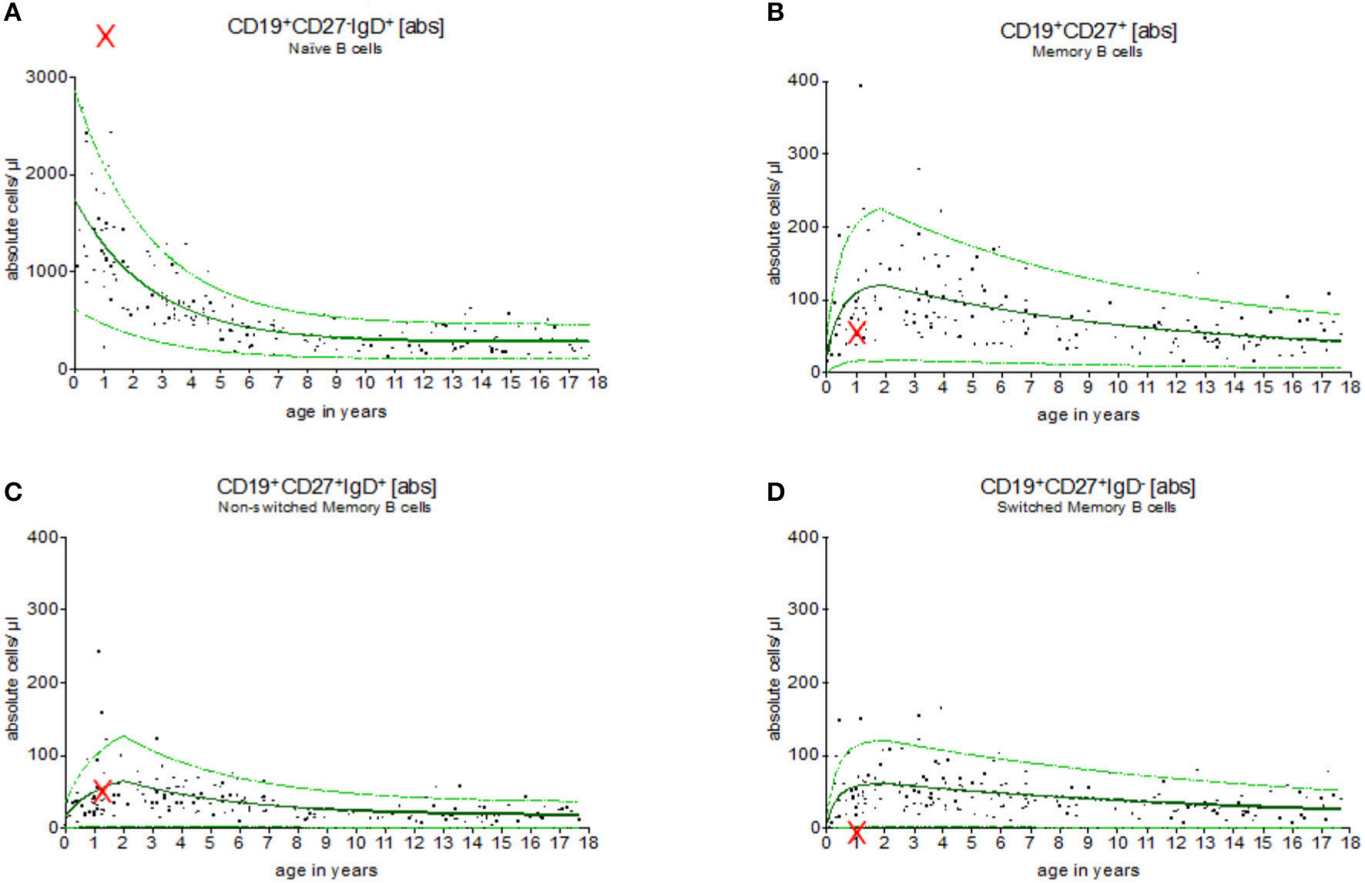
Evaluation of B cell subsets in a pediatric patient with CD40 ligand deficiency syndrome. Using the exponential regression model upper and lower normal range levels and an age-depended predicted value are provided. The 7 months old boy showed a complete lack of switched memory B cells **(D)** while naïve **(A)**, memory **(B)** and non-switched memory B cells **(C)** were within the normal range (indicated as “x”). These results were in line with the diagnosis of CD40 ligand deficiency, which was then functionally and genetically confirmed.

Figure 5 provides an example for the use of the exponential model of B cell subsets in the evaluation of children with primary immunodeficiencies. A 7 months old boy was assessed showing a complete lack of B cell switch with upregulated level of naïve, and normal levels of memory, and non-switched B cell counts in peripheral blood. The result suggested a CD40 ligand deficiency, which was further confirmed functionally on patients activated T cells and genetically (a large deletion in CD40L gene).

## Discussion

Diagnostics, including B lymphocytes numbers and distribution from infancy to adulthood, require the application of age-matched reference values for the interpretation of flow cytometric B cell analysis in children. B cell development, like the development of other cell types of the immune system, is a dynamic process (32, 33). However, limited data is available on the dynamic development of age-grouped reference values. In this study, we present a continuous exponential regression model using a rapid and simplified five-color flow cytometric analysis, where CD27 and IgD in combination with CD19 or CD20 were applied to divide B cells into CD27^−^IgD^+^ naïve, CD27^+^IgD^+^ non-switched memory and CD27^+^IgD^−^ switched memory B cells. In this analysis, we observed a decrease in the naïve B cell pool until the age of ten, before stabilization. Non-switched, switched and memory B cells increased notably within the first 5 years and remained stable thereafter. There was no significant difference in B cell numbers when CD19 was used for total B cell numbers compared to CD20.

Morbach et al. utilized a generalized additive model estimating a smoothing spline (34) via non-parametric regression. A reduction of total B cells and transitional B cells in PB was shown, whereas the fraction of switched and non-switched memory B cells increased gradually during the first 5 years of life and remained stable thereafter. Other B cell subpopulations, such as CD21^low^CD38 ^low^ B cells or plasmablasts, did not undergo age-related development (31). Duchamp et al. carried out a national multi-centric study in France, analyzing naïve, switched and non-switched memory B cells in 7 age groups, including 242 pediatric patients. Absolute numbers and the percentage of B cell subpopulations as well as median values with the corresponding 5th and 95th percentiles were provided. Their results were in line with Morbach et al., confirming the dynamics of age-dependent B cell development. The authors postulated that the increase of non-switched memory B cells might reflect the immaturity of the T-cell-independent antibody production after pathogen encounters during the first years of life. Duchamp et al. analyzed total CD19+ peripheral B cells and their CD27 and IgD expression (28), whereas Morbach et al. analyzed further subpopulations by CD38, CD21 and CD 24 expression (31). These studies did not include CD20 expression.

In comparison to most other studies, in our study, no anticipated age groups were used, but continuous models were generated based on an exponential regression analysis.

Especially with regard to identifying pediatric patients with PIDs and understanding the pathophysiology of novel monogenic PIDs, the access to age-matched reference values is of paramount importance. While B cell subpopulations have been intensively studied in patients suffering from “classical” CVID, the role of B cells and the detailed B cell profile in recently described monogenic PIDs, such as LRBA- deficiency or CTLA-4 haplo-insufficiency, have not been completely understood. Recent data show that unlike “classical” CVID, these disorders can severely affect very young children. For LRBA- deficiency, it was recently shown that only a subgroup of patients suffered from infectious complications and hypogammaglobulinemia. The majority of the affected patients developed severe autoimmunity as their leading symptom affecting multiple organs, which remained refractory to immunosuppressive treatment (35).

The detailed analysis of B-cell subsets is crucial to understand the underlying pathology of severe autoimmune disorders in these patients and other individuals suffering from primary or secondary autoimmune disorders. Furthermore, B cells represent a therapeutic target for the treatment of refractory autoimmune and malignant B cell disease. Eliminating B cells using rituximab as an anti-CD19 antibody has been used in recent years in the treatment of autoimmunity (24). Recent data suggest that the elimination of plasma cells using bortezomib or daratumumab may be beneficial in the treatment of refractory autoimmunity in children (25). Furthermore, a close monitoring of B cell engraftment post alloHSCT and B cell subset reconstitution after anti-cellular treatment is of utmost importance.

The continuous reference values presented herein were integrated in the local laboratory information system. Throughout this tool, determined B cell values were automatically correlated to reference ranges and highlighted if abnormal. Aberrations in the B cell subpopulation are easily noticeable by the clinician as a fundamental prerequisite for clinical diagnostics, thus providing a substantial benefit for patient diagnosis and following treatments. This model can easily be expanded for further B cell subtypes, especially for several Breg subsets, which might provide insight into the underlying condition of patients suffering from autoimmunity.

## Author contributions

CK, SH, SS-S, and SB provided data, analyzed and wrote the manuscript. AQ, CC, UK, MB, CK, performed the experiments and analyzed the data. PB, UR, TK, KB, DS provided clinical data and patients samples.

### Conflict of interest statement

The authors declare that the research was conducted in the absence of any commercial or financial relationships that could be construed as a potential conflict of interest.
